# The Mechanical Characterization of Welded Hybrid Joints Based on a Fast-Curing Epoxy Composite with an Integrated Phenoxy Coupling Layer

**DOI:** 10.3390/ma15031264

**Published:** 2022-02-08

**Authors:** Lucian Zweifel, Klaus Ritter, Christian Brauner

**Affiliations:** 1 Institute of Polymer Engineering, University of Applied Sciences and Arts Northwestern Switzerland (FHNW), Klosterzelgstrasse 2, 5210 Windisch, Switzerland; christian.brauner@fhnw.ch; 2 Huntsman Advanced Materials, Klybeckstrasse 200, 4057 Basel, Switzerland; klaus_ritter@huntsman.com

**Keywords:** phenoxy, welding technology, interphase, ultrasonic welding, weld properties, advanced composites

## Abstract

The joining of composites mostly relies on traditional joining technologies, such as film or paste adhesives, or mechanical fasteners. This study focuses on the appealing approach of using standard thermoplastic welding processes to join thermosets. To achieve this, a thermoplastic coupling layer is created by curing with a thermoset composite part. This leads to a functional surface that can be utilized with thermoplastic welding methods. The thermoplastic coupling layer is integrated as a thin film, compatible with the thermoset resin in the sense that it can partially diffuse in a controlled way into the thermoset resin during the curing cycle. Recent studies showed the high affinity for the interphase formation of poly hydroxy ether (phenoxy) film as coupling layer, in combination with a fast-curing epoxy system that cures within 1 min at 140 °C. In this study, an investigation based on resistance and ultrasonic welding techniques with different testing conditions of single-lap shear samples (at room temperature, 60 °C, and 80 °C) was performed. The results showed strong mechanical strengths of 28.9 MPa (±0.7%) for resistance welding and 24.5 MPa (±0.1%) for ultrasonic welding, with only a minor reduction in mechanical properties up to the glass transition temperature of phenoxy (90 °C). The combination of a fast-curing composite material with an ultra-fast ultrasonic joining technology clearly demonstrates the high potential of this joining technique for industrial applications, such as automotive, sporting goods, or wind energy. The innovation allowing structural joining performance presents key advantages versus traditional methods: the thermoplastic film positioning in the mold can be automated and localized, joint formation requires only a fraction of a second, and the joining operation does not require surface preparation/cleaning or structure deterioration (drilling).

## 1. Introduction

Reliable and cost-effective joining technologies for fiber-reinforced composite materials provide a great potential to significantly reduce weight, fuel consumption, and, consequently, CO_2_ emissions [1,2,3,4,5]. Therefore, it is essential to develop and implement new joining technologies to further improve the manufacture and assembly of structural composite parts. The use of thermoplastic joining processes has certain benefits in joining thermoset composite structures. So far, joining similar and dissimilar composite has mostly relied on traditional joining technologies such as adhesive bonding or mechanical fasteners, which both have distinct drawbacks. Joining with adhesives results in high costs and long process times, whereas mechanical fasteners introduce weakness into the structure (stress concentrations) due to the necessity of drilling holes, where the continuous fiber reinforcement is disrupted. In contrast, thermoplastic welding offers the unique ability of melting and reprocessing compared to thermosets, which cannot be re-melted after cross-linking. Additionally, thermoplastic welding allows for fast processing speeds without significant surface preparation efforts, resulting in strong and dependable mechanical performance [4,6].

**Figure 1:**
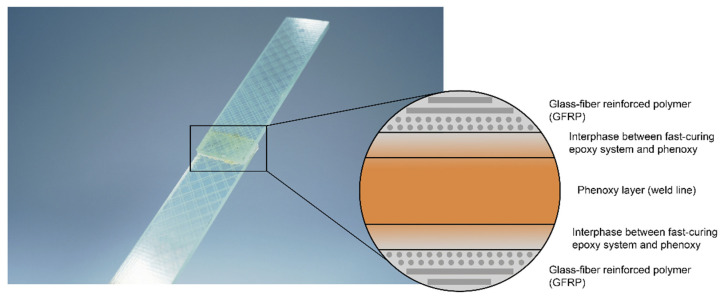
Concept of weldable hybrid joints based on a fast-curing epoxy composites with an integrated poly hydroxy ether (phenoxy) coupling layer.

Within the last three decades, thermoplastic welding processes have become increasingly interesting in research, as well as for use in industry moving towards primary structures in aerospace [1]. Here, resistance welding [6,7,8,9,10,11,12], induction welding [1,2], ultrasonic welding [6,13,14,15,16], and laser welding [6] have proven their potential as reliable joining technologies. Since 2001, the glass fiber-reinforced polyphenylene sulfide (PPS) J-Noses for the Airbus A340-500/600 and A380-800 are resistance-welded in series production, resulting in a weight reduction of greater than 20% [17,18]. In 2010, Fokker Aerostructures designed and developed the Gulfstream G650 tail section and industrialized the induction welding method for the rudder and the elevator using carbon fiber-reinforced PPS, reducing the weight of the components by 25% compared to traditional materials [12,19]. Within the EU’s CleanSky 2 program, ultrasonic welding was utilized to spot-weld short fiber-reinforced thermoplastic clips to the skin and stringers of a multifunctional fuselage demonstrator [20,21]. Furthermore, MM-Welding^®^ has presented innovative fastening techniques to create cost-effective joining of fixation elements in differently structured materials (porous materials, sheet structures, sandwich materials, injection-molded materials, and pressure moldings) via ultrasonic energy in the automotive field [22,23]. Recently, robotic-based, continuous welding of thermoplastic structures was introduced at DLR Augsburg [24], showing the potential towards the energy-efficient automation of welding processes. Each technology has shown distinct limitations, such as resistance welding often using a metal wire mesh, which is ideal for insulating materials, such as glass fibers, but leads to energy leakage with carbon-reinforced composites due to conductivity [12]. The above-mentioned applications focused on thermoplastic composites for high-performance applications using materials such as fiber-reinforced PPS, polyetheretherketone (PEEK), polyetherketoneketone (PEKK), polyaryletherketone (PAEK), and polyetherimide (PEI). However, an interesting question is whether it is possible to also apply welding to dissimilar composites, e.g., thermoplastic and thermoset composite combinations, as a composite-friendly alternative to current assembling procedures. Here, the attractive concept of using the thermoplastic welding process for thermosets was introduced [25] by including a thermoplastic-rich layer during the curing process of a thermoset composite. The so-called thermoplastic coupling layer acts as a functionalized, or ‘weldable’, surface (see Figure 1).

The affinity between thermoset and thermoplastic is the crucial element for creating joints with high strength [13,26,27,28]. A gradient interphase forms between the reactive epoxy resin and the thermoplastic material, whereby the two components partially dissolve, diffuse, and, finally, decompose due to a reaction-induced phase separation [29,30,31]. The overall composition or cross-link density of the material varies from location to location at the macroscopic level [32]. The decomposed morphology enables strong mechanical interlocking for subsequent load transfer [28]. Recent studies have proposed PEI as a suitable candidate for interphase formation in aerospace-related applications [9,13,26,28,30,33,34,35]. In a previous study, an identical concept was applied to automotive-related applications with a fast-curing epoxy system (Araldite LY3585/Aradur 3475) [27]. It was possible to establish an interphase between poly hydroxy ether (phenoxy) and the fast-curing epoxy system within a fraction of a minute, leading to a high mechanical performance of single-lap shear samples joined by resistance welding, with an average strength of 25.3 MPa [27]. For automotive applications, comparable structural adhesives are listed in Table 1. The comparison between structural adhesive bonding and thermoplastic welding strengthens the potential of the latter. Therefore, the joining of thermosets via a phenoxy boundary layer is a reliable, cost-efficient technique, which offers the possibility of creating lightweight design concepts.

Within this study, an investigation using a fast-curing epoxy resin and phenoxy was conducted with resistance and ultrasonic welding techniques whereby the lap shear strength (LSS) was evaluated using single-lap shear tests with different testing conditions (at room temperature, 60 °C, and 80 °C). In addition, the failure mechanism of fractured samples combined with optical microscopy of the microstructure was analyzed. The objective was the adaptation from lab-scale trials to industrial-scale manufacturing methods utilizing dynamic fluid compression molding (DFCM), which is an efficient compression molding process whereby a one-shot phenoxy film integration was tested.

## 2. Materials and Methods

### 2.1. Materials

**Figure 2:**
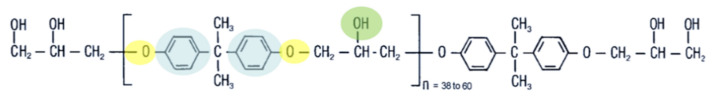
The polymer structure of poly hydroxy ether (phenoxy).

Phenoxy polymers are commercially available in different forms such as emulsion, powders, films, and granulates. In this study, the phenoxy grade PKHH (Huntsman Advanced Materials, Basel, Switzerland), a grade for extrusion, was selected as the most promising candidate for welding processing. The structure of this polymer (Figure 2) shows amorphous thermoplastic properties, such as rigidity, thermal and chemical stability, and adhesion strength. The rigidity and thermal stability result from the aromatic compounds (marked in blue), the chemical stability of the oxygen atoms in the main chain (marked in yellow), and the adhesion strength of the hydroxyl groups (marked in green). The PKHH material has a molecular weight of 52,000 g/mol [40], and compared to available phenoxy resins, such as PKHB (32,000 g/mol) and PKFE (60,000 g/mol), it is positioned in the upper range [40]. Two different film thicknesses (75 µm and 125 µm) were used for parametric studies and were supplied by Huntsman Advanced Materials, Basel, Switzerland.

In this study, an epoxy resin system (Araldite LY3585/Aradur 4375) provided by Huntsman Advanced Materials (Basel, Switzerland) was used. The system is widely applied in the mass production of structural automotive components and is notable for its short curing cycles and final glass transition temperature of approximately 120 °C. The mentioned system is typically used for high-pressure resin transfer molding (HP-RTM), wet compression molding, and dynamic fluid compression molding (DFCM) applications, with a curing cycle of 2 min at 115 °C and 1 min at 140 °C, respectively. The DFCM process combines the speed of wet compression molding with the quality of HP-RTM without sacrificing geometric complexity [41,42]. With the usage of vacuum and dynamic mold pressure, void-free impregnations are achieved [41]. The epoxy resin system is based on bisphenol-A-diglycidylether as resin and 1,3-cyclohexanedimethanamine and methyldiethanolamine as hardeners. A kinetic model has been developed by the authors using the modified Kamal-Sourour model to describe the curing process [27], which will also be used in this study.

### 2.2. Manufacturing Methods

Composite plates were manufactured using the DFCM process. The plates were cured for 1 min at 140 °C, resulting in a part production time of 1.5 min. The process consisted of the following steps: First, the mold was heated to 140 °C. Second, the defined amount of resin mix was spread onto the preform, outside of the mold. Third, the preform (with the resin mix on top) was placed into the mold. Fourth, the mold was partially closed, and the vacuum was drawn. After 10 s, the mold was fully closed, and after an additional 60 s of curing with an applied pressure of 30 bars, the mold was opened, and the part was removed. The laminate consisted of two main types of fabrics: two layers of a canvas weave, glass fiber fabric with an aerial weight of 200 g/m^2^ (0/90, Porcher Industries, Erbach, Germany) as outer layers, and six layers of a glass fabric with a higher aerial weight of 600 g/m^2^ (EBX 600, 45/−45, Saati Composites, Appiano Gentile, Milano, Italy) as inner layers. Thus, two different layups were used, with the labeling L1 (0/−90_GF200_, 45/−45_GF600_, −45/45_GF600_, and 45/−45_GF600_)_s_ and L2 (45/−45_GF600_, −45/45_GF600_, and 45/−45_GF600_)_s_. Glass fiber fabrics were selected because they allow a clear visual inspection of the sample after manufacturing, joining, and testing. Different types of fabrics were chosen, as the weave pattern significantly influences crack propagation and LSS [7,43]. The mold dimension was 530 × 530 mm^2^, which theoretically results in approximately 95 single-lap shear coupons.

**Figure 3:**
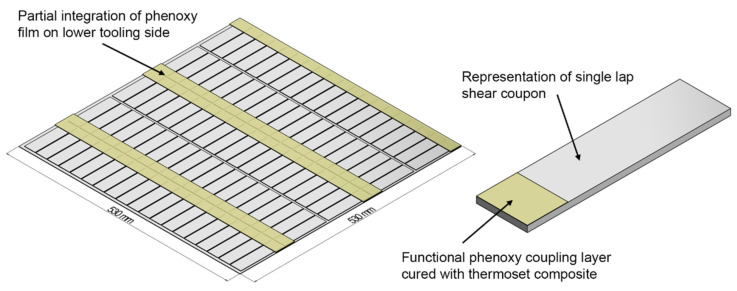
Schematic lap shear coupon array with partial integration of the poly hydroxy ether (phenoxy) film on the lower tooling side.

The sensitive element of the manufacturing was the introduction of the thermoplastic film into the process in a reliable, robust way that could be later adapted by industrialization. Thus, the thermoplastic coupling layer was added on the lower side of the tool with a thickness of 125 μm, which provides material for interphase formation (approximately 13 μm at a 100 °C curing temperature [27]) and welding. It was assumed that the considerably small thickness of the phenoxy film does not influence the residual stress of the laminate stacking sequence. The phenoxy film was partially integrated in joining areas (Figure 3) at positions where an actual welding was later conducted (applying the right material in the right place). In this study, the phenoxy film was directly taped into the mold before the mold was heated up.

### 2.3. Welding and Test Methods

Two welding methods, namely, resistance welding and ultrasonic welding, were used and compared in this study. The welded joints were mechanically tested following the ISO 4587 standard using a Zwick 100 kN universal tensile test machine (Zwick Roell, Ulm, Germany) with a testing speed of 2 mm/min to determine the apparent LSS. The LSS was calculated as the maximum load divided by the overlap area. No surface preparation of the overlapping area was applied. As phenoxy grades are sensitive to water absorption, the samples were dried for 5 h at 80 °C [42]. Furthermore, fractured specimens were analyzed by visual examination of the fracture area according to DIN EN ISO 10365. First, a comparative study was performed with resistance and ultrasonic welding, whereby the process parameters were iteratively optimized to achieve maximum mechanical properties. Here, both welding methods have been assessed by determining of the LSS at room temperature, followed by an analysis of the fractured area. Second, the ultrasonic-welded samples were tested at higher temperatures (at 60 °C and 80 °C). The testing temperatures were chosen due to their relevance in the automotive fields, as well as to test the limit of mechanical performance below the glass transition temperature of phenoxy (~91 °C). For each parametric configuration, five samples were welded and tested.

#### 2.3.1. Resistance Welding

The custom-built resistance welding setup, developed in a previous study [8], was used to join single-lap shear test specimens. The welding process parameters were experimentally optimized in a preliminary study resulting in a power of 25 kW/m^2^, a pressure of 1 MPa, and a total welding time of 80 s, followed by 180 s of cooling time while the pressure was maintained. Detailed investigations of the process parameters coupled with in-situ process monitoring were described in [8,27]. Further, a stainless steel heating element with a wire diameter of 36 μm, a mesh size of 50 μm, and an open area of 33.8% was used [27]. The heating elements were cut to dimensions of 12.5 mm wide and 50 mm long.

#### 2.3.2. Ultrasonic Welding

In this study, a pneumatic-driven ultrasonic welder (Branson 2000Xc, Emerson Automation Solutions, Baar, Switzerland) was used. The equipment monitored force, displacement, amplitude, frequency, and energy, with a sampling rate of 100 Hz. A rectangular sonotrode made of titanium alloy (Ti-6Al-4V) with a welding area of 19 × 38 mm^2^ was used. The sonotrode has a gain factor of 1.5, resulting in a maximum amplitude of 76 µm at 100% power output. The optimal process settings for ultrasonic welding were experimentally determined in a preliminary study, resulting in the following parameters: trigger force, 50 N; ultrasonic active, 0.5 s; amplitude, 100%; weld pressure, 250 kPa; hold pressure, 350 kPa; hold time, 2 s; and operating frequency, 20 kHz. The hold process after welding was important to ensure an ideal consolidation quality by cooling the joining area.

One method to generate controlled heat in the welding zone is to introduce energy directors in the ultrasonic welding process to concentrate the heat generation within the boundary of the two constituents through a combination of surface friction and viscoelastic heating [44]. For all the welded joints, a 75 μm thick, flat phenoxy energy director was used to concentrate heat generation at the welding interface. Flat energy directors are neat resin films with a slightly larger size of 30 × 30 mm^2^ compared to the joining area. The films were placed between the adherends before welding. Flat energy directors led to similar results compared to more traditionally molded energy directors [13,45,46,47]. Recent studies analyzed energy director-less joints compared to flat energy directors, whereby a significant reduction of LSS was apparent [33]. Furthermore, the influence of the final weld line thickness between the two adherends on the resulting LSS is significant, and it is comparable to traditional adhesive technology. It has been shown experimentally by many authors that the joint strength decreases as the weld line thickness increases due to the introduction of bending stresses [7,48,49]. Consequently, there is an optimum value of weld line thickness for which the LSS is maximized. Therefore, a second energy director thickness of 125 μm was used for one configuration of ultrasonic welding.

## 3. Results and Discussion

### 3.1. Analysis of Phenoxy Film Integration during Manufacturing

**Figure 4:**
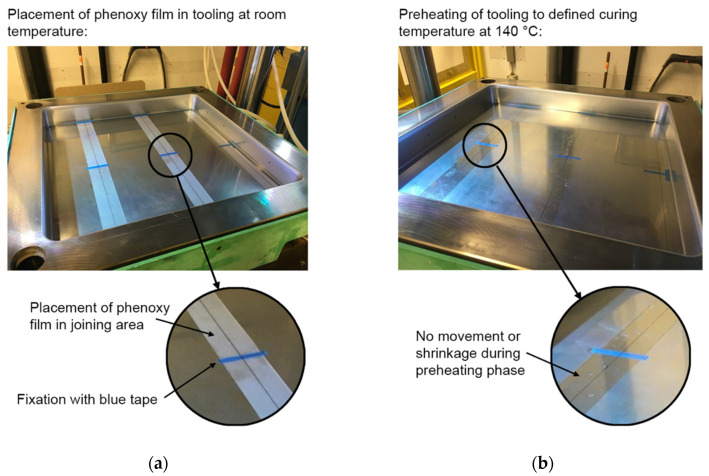
One-shot phenoxy film integration during the preheating phase of the dynamic fluid compression molding (DFCM) process. (**a**) Placement of the phenoxy film in the tooling at room temperature. (**b**) Preheating of the tooling to a defined curing temperature of 140 °C.

Figure 4 presents the concept of the one-shot phenoxy film integration before and after the preheating phase of the DFCM process. The fixation of the film was achieved by using a temperature-resistant tape that secured any movement in plane direction. During the heat up of the mold to the temperature of 140 °C for the DFCM process, the film was stable in position, without shrinkage effects (see Figure 4b). Furthermore, no overflow of resin on the welding surface was visible.

**Figure 5:**
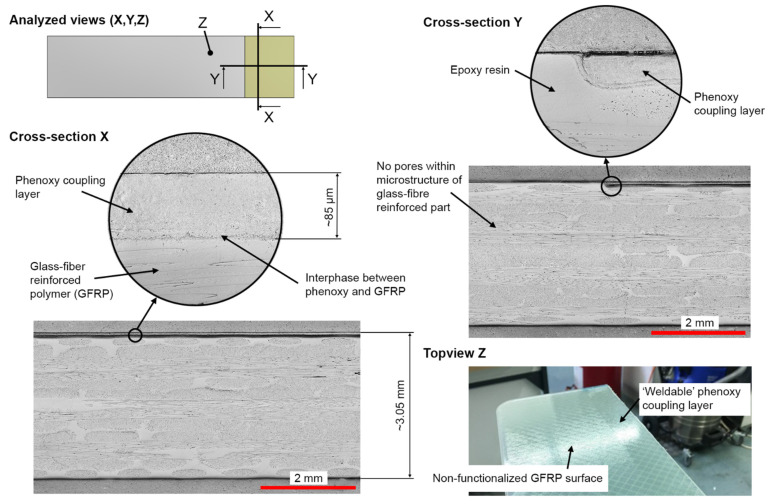
Cross-sectional optical micrographs and visual appearance of the phenoxy/GFRP plate samples: X: Optical representation of the phenoxy coupling layer showing a high affinity to the epoxy resin; Y: Resulting transition between the cured epoxy resin and the integrated phenoxy coupling layer; Z: Visual appearance of the ‘weldable’ and ‘non-weldable’ surface on a phenoxy/GFRP sample.

After curing the composite plate, the phenoxy film was fully integrated in the thermoset glass fiber-reinforced polymer (GFRP) part, showing no indication of separation and no change in position or distortion from the vacuum, and therefore demonstrating the high affinity of the two components [27]. Despite the high curing temperature, and thus the fast polymerization of the thermoset within less than 1 min, the phenoxy film partially dissolved and formed an interphase with the epoxy resin, as shown in [27] with Raman spectroscopy. The actual time for interphase formation is much less than 60 s, as the highest diffusion rate prevails in the beginning of the curing. As the chain mobility reduces with a higher degree of curing, the diffusion rate drops rapidly, eventually leading to the maximum interphase thickness within the first 10 s of curing [28,30,31,50]. Voleppe et al. [51] showed that the penetration at the front of the thermoset continued beyond phase separation, whereby both mechanisms overlapped. The measurement of the phenoxy coupling layer was 85 μm after curing (see Figure 5), whereas the nominal film thickness prior to curing was 125 μm. The actual thickness after curing cannot be measured by optical microscopy as the contrast between the two components is not sufficient to provide clear distinction due primarily to their similar chemical structure, as both phenoxy and the epoxy systems are based on bisphenol A, which results in a similar optical appearance and spectroscopy response [27]. The layup resulted in an average laminate thickness of 3.05 mm with a standard deviation of ±3%. The quality was consistent for all four manufactured plates, with the absence of porosity and a constant fiber volume content. The cross-sections shown in Figure 5 present the similar appearances of the interphase shown in [27]. Furthermore, no porosities were found within the phenoxy/GFRP interphase.

### 3.2. Welding and Evaluation of Mechanical Performance

**Figure 6:**
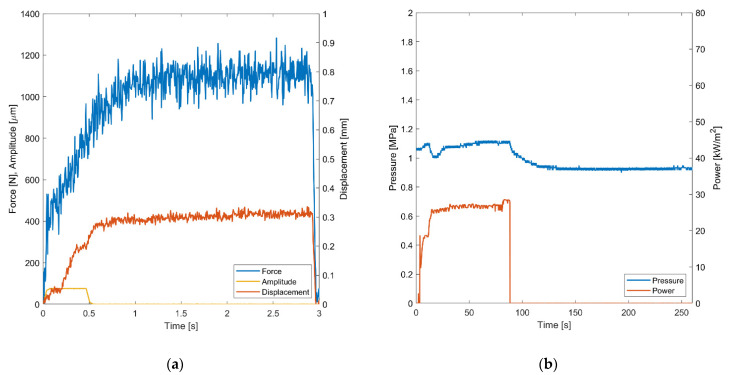
Comparison of online process monitoring for both thermoplastic welding processes. (**a**) Force, amplitude and displacement of the designated ultrasonic welding process sampled at 100 Hz. (**b**) Pressure and power of the designated resistance welding process sampled at 100 Hz.

In the following section, the welding results are presented. First, exemplary online process monitoring results for the ultrasonic (Figure 6a) and resistance welding (Figure 6b) processes are shown to provide insight into the welding process. In a second step, the visual appearance is compared. Third, the results of the mechanical tests are shown by analyzing the fracture surface, shear stress-displacement curves, and the resulting LSS. For the resistance welding, a welding pressure of 1 MPa was selected. During the process, the phenoxy was heated by the Joule effect [9,10,11]. After reaching the glass transition temperature of approximately 90 °C, the phenoxy interlayer starts to soften. Reaching the glass transition point is visible in a drop of applied pressure after 20–30 s. The total welding time was 80 s, with a further consolidation during cooling for 180 s. Therefore, the total process time was around 260 s. In comparison to the resistance welding process, the ultrasonic welding process was faster with a total time of 3 s. In this case, the welding process takes 0.5 s using an amplitude of 76 µm, followed by a consolidation time of 2 s.

**Figure 7:**
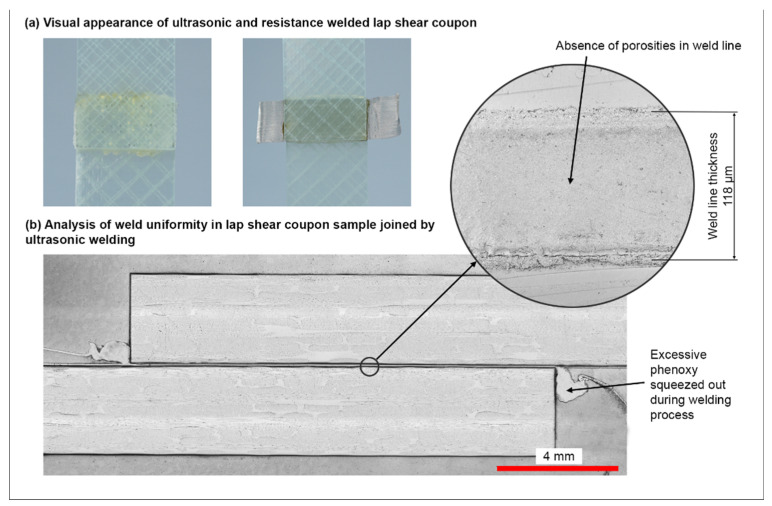
(**a**) Visual appearance of the ultrasonic- and resistance-welded lap shear coupons. (**b**) Micro-polished cross-section of lap shear coupon joined by ultrasonic welding.

Both welding technologies were optimized successfully with proper visual appearance of the samples (see Figure 7a). Only a slight flow of the thermoplastic material was apparent for both resistance and ultrasonic welding. For ultrasonic welding with the 75 μm thick energy directors, the final weld line thickness was approximately 118 μm with a standard deviation of 7 μm (±6.5%). Thereby, the weld line quality was satisfactory and consistent, with only minor porosities for samples joined by ultrasonic welding, and it was comparable to other research [13,52]. The micrographs underline the ideal process parameters and settings to exploit the full potential of the joining technology. Single-lap shear joints are prone to peel stresses and excessive shear stress on the tip of the weld line [49,53]. Consequently, excessive material that was squeezed out of the joining area during welding was removed before testing, primarily due to maintaining a consistent joint design throughout the joined coupons because the fillets formed by the squeeze flow did not show the exact characteristic for each weld performed.

**Figure 8:**
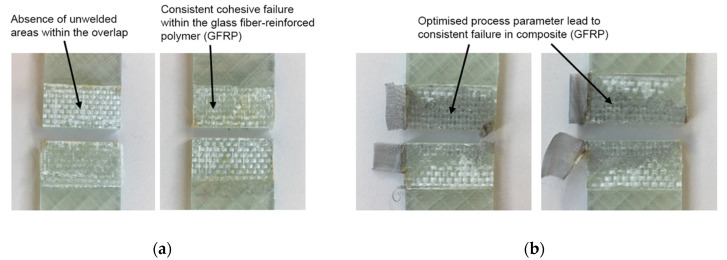
Exemplary fractured samples for (**a**) ultrasonic welding and (**b**) resistance welding.

The samples were tested to derive the LSS. In Figure 8, the tested samples are presented in detail to highlight the different failure types. As visible, the failure types were consistent for all samples where the failure occurred mostly within the GFRP (adherend) and not as an adhesive or cohesive failure. This confirms that the process parameters were selected properly for both welding methods. In the case of too low temperatures, this would lead to a cohesive failure between the samples. In the case of too high temperatures, this would lead to a color change (thermal degradation) of the welded zone and to an adhesive failure. In the thermogravimetric analysis measurement published in [54], the thermal stability of phenoxy was characterized by showing an onset of decomposition at 340 °C. It was observed from the results that the dominant composite failure is the ideal case because the joining area is stronger than the composite itself. Figure 7 supports this argument, as no porosities or other defects were present in the optical micrograph of the sample cross-section. Furthermore, there were only minor unwelded areas detected within the overlap, which eventually explains the drop in LSS of some samples at higher testing temperatures.

**Figure 9:**
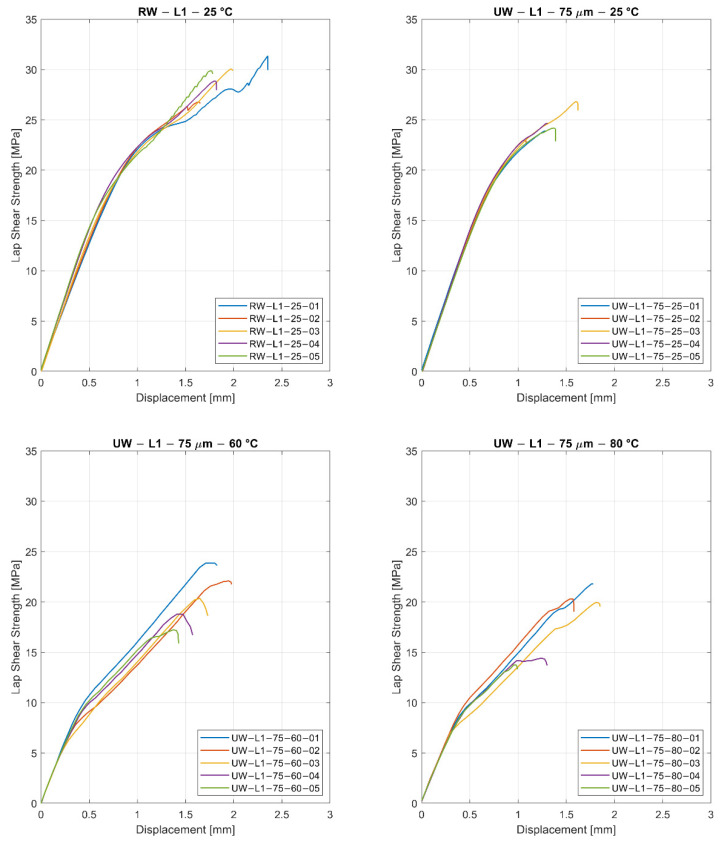
Comparison of stress versus displacement depending on the welding method (RW = resistance welding and UW = ultrasonic welding), layup, energy director, and testing temperature.

In Figure 9, the shear stress-displacement curves are shown for the two welding methods. It is visible that both welding methods show a linear, consistent behavior until 15 MPa. After this, failure of the specimens starts. The typical failure is not brittle, and it seems that the damage tolerance is higher compared to the typical failure of an adhesive-bonded specimen [48,49].

Table 2 illustrates the average LSS between two welded phenoxy/GFRP coupons with their respective thermoplastic boundary layers for resistance welding (RW) and ultrasonic welding (UW). Using resistance welding, the maximum shear strength was 28.5 MPa with a standard deviation of 2.3 MPa. The comparable ultrasonic welded specimens (L1, 75 μm, 25 °C) failed at 23.8 MPa with a standard deviation of 0.6 MPa. In comparison, the resistance-welded samples have a 19% higher strength, which was explained by the incorporation of the steel mesh that acts as a reinforcement. The mesh improves the shear strength because it influences the crack propagation, whereby micro-cracks are deflected [7]. As is visible in the results, the thickness of the phenoxy layer (energy director) has only a minor influence on the shear strength, especially because the final weld line thickness is more influenced by the pressure applied during the weld (with dependency on squeeze flow). Both 75 μm and 125 μm energy directors led to a similar weld line thickness between 110–120 μm. In [27], it was shown by hot stage microscopy and Raman microscopy that for isothermal curing conditions at 100 °C with similar materials results in an interphase with a thickness of 13 μm with a linear concentration gradient. Furthermore, the change in layup (L1, L2) shows a significant drop in apparent LSS as the propagation of micro-cracks depends on the weave pattern located on the outer layer of the composite.

In a second step, ultrasonic-welded samples were tested at 60 °C and 80 °C. The glass transition temperature of the utilized phenoxy grade PKHH is 91 °C (further data about the temperature-dependent stiffness was published in [54]). In [54], three-point bending tests using a DMA Q800 (Dynamic Mechanical Analysis) were performed on a phenoxy PKHB sample. The results have shown a very linear decrease of the storage modulus until the glass transition onset of 91 °C was reached [54]. The resulting LSS was influenced by the temperature but remained at a considerable high strength, with minor reductions for 60 °C (3.4%) and 80 °C (12%). Here, small defects within the welding interface, such as unwelded areas or weld line thickness inconsistency, became more severe and eventually resulted in a higher scattering of the apparent maximum LSS. In comparison to the tests at room temperature, the displacement at maximum shear strength slightly increased at higher testing temperatures due to the more ductile behavior of the welding area. Furthermore, it was possible to allocate small, unwelded areas within the samples ‘UW-L1-75-80-04’ and ‘UW-L1-75-80-05’, which provides a reasonable explanation for the drop in maximum LSS compared to the other samples tested at 80 °C.

## 4. Conclusions

In this paper, the experimental assessment of the manufacturing and mechanical characterization of phenoxy/GFRP hybrid joints based on ultrasonic and resistance welding was presented. Besides the successful phenoxy film integration during manufacturing, different welding configurations were analyzed: influence of welding method on mechanical performance, effect of weave pattern on lap shear strength (LSS), and reduction of LSS with increased temperature. The analysis of the results can be summarized as follows:

- Integration of the phenoxy coupling layer in the composite structure was carried out in one shot during the used compression molding process, which means no additional energy or process step is required. The integration of the film was robust and, even in the compression-molding process with high-pressure gradients, no movement of the film occurred. Furthermore, no overflow of resin on the welding surface was visible and no visible shrinkage of the film during heating to the curing temperature of 140 °C appeared.
- In this study, it was presented that a strong connection between phenoxy and epoxy resin can be reached, even for very fast curing systems, with a curing time of 1 min at 140 °C.
- Lightweight joining technology with a robust ultrasonic welding process, a high average LSS of 24.4 MPa, and a standard deviation of 0.4 MPa have been achieved. The process time for welding was about 3 s, which is remarkable for the joining of a thermoset composite part. Based on the very linear storage modulus dependency on temperature of the phenoxy until the glass transition temperature (onset of 91 °C), the LSS of samples tested at 60 °C and 80 °C showed considerably high LSS results of 20.1 MPa and 18.3 MPa, respectively.
- Very short welding process times of 3 s for ultrasonic welding and 260 s for resistance welding with damage-tolerant joint design were reached, in comparison to state-of-the-art, epoxy-based adhesives. The welding process is surface-tolerant, which means no preparation is necessary. Furthermore, the proposed joining technology can be easily controlled and automated, and it is therefore adaptable for mass production.
- The use of thermoplastic as a joining material reduces the exposure of workers to chemicals (reactive adhesives). Furthermore, the thermoplastic coupling layer reduces the overall weight in comparison to mechanical fasteners and allows de-assembling possibilities. Due to all these factors, the overall environmental impact is reduced.

The performed study highlights the potential of welded hybrid joints based on a fast-curing epoxy composite with an integrated phenoxy coupling layer, as well as their application in the fields of general transport, wind energy, or sporting goods. In addition to the achieved results, there remain points that should be addressed in further studies, such as the long-term effects on fatigue, creep behavior, and humidity, to clarify the full potential of these weldable hybrid joints.

## Figures and Tables

**Table 1:** Comparison of commercial structural adhesive solutions utilized in the automotive industry (GFRP = glass fiber-reinforced polymer; CFRP = carbon fiber-reinforced polymer, LSS = lap shear strength).

Structural Adhesive	Curing Condition	Adherend	Testing Temperature	LSS (MPa)
SikaPower^®^ 1200 [36]	4 h at 70 °C	GFRP	23 °C	20
3M™ Structural Adhesive SA9820 [37]	24 h at RT followed by 30 min at 170 °C	Aluminum	23 °C	20
3M™ Structural Adhesive SA9820 [37]	24 h at RT followed by 30 min at 170 °C	Aluminum	80 °C	13
Araldite^®^ 2012 [38]	16 h at 40 °C	CFRP	23 °C	14.5
SikaPower^®^-1277 [39]	2 weeks at 23 °C	Steel	23 °C	28

**Table 2:** Resulting lap shear strength (LSS) depending on welding technology (RW = resistance welding, UW = ultrasonic welding), layup, energy director, and testing temperature.

Welding	Layup	Energy Director	Testing Temperature	LSS (MPa)	Standard Deviation (MPa)
RW	L1	-	23 °C	28.5	2.3
UW	L1	75 μm	23 °C	23.8	0.6
UW	L1	125 μm	23 °C	24.4	0.4
UW	L2	75 μm	23 °C	20.8	2.2
UW	L1	75 μm	60 °C	20.1	2.4
UW	L1	75 μm	80 °C	18.3	3.3

## Data Availability

The data presented in this study are available on request from the corresponding author.

## References

[1] Favaloro M. Thermoplastic Composites in Aerospace–The Future Looks Bright. https://www.compositesworld.com/articles/thermoplastic-composites-in-aerospace-past-present-and-future.

[2] Starke J. Carbon Composites in Automotiv Structural Applications. https://cutt.ly/CteyLEP.

[3] Meng F., Pickering S.J., Mckechnie J. An Environmental Comparison of Carbon Fibre Composite Waste End-of-life Options. Proceedings of the SAMPE Europe Conference.

[4] Gardinger G. New Horizons in Welding Thermoplastic Composite. https://www.compositesworld.com/articles/new-horizons-in-welding-thermoplastic-composites.

[5] Wegmann S., Rytka C., Diaz-Rodenas M., Werlen V., Schneeberger C., Ermanni P., Caglar B., Gomez C., Michaud V. (2022). A Life Cycle Analysis of Novel Lightweight Composite Processes: Reducing the Environmental Footprint of Automotive Structures. J. Clean. Prod..

[6] Gardinger G. Welding Thermoplastic Composites. https://www.compositesworld.com/articles/welding-thermoplastic-composites.

[7] Shi H., Villegas I.F., Bersee H.E.N. (2013). Strength and Failure Modes in Resistance Welded Thermoplastic Composite Joints: Effect of Fibre-Matrix Adhesion and Fibre Orientation. Compos. Part A Appl. Sci. Manuf..

[8] Zweifel L., Brunner J., Brauner C., Dransfeld C. Development of a Resistance Welding Process for Thermoset Fiber Composite Components with Co-Cured Thermoplastic Boundary Layer. Proceedings of the European Conference on Composite Materials.

[9] Ageorges C., Ye L. (2006). Resistance Welding of Thermosetting Composite/Thermoplastic Composite Joints. Adv. Mater..

[10] Stavrov D., Bersee H. (2005). Resistance Welding of Thermoplastic Composites-An Overview. Compos. Part A Appl. Sci. Manuf..

[11] Ageorges C., Ye L., Hou M. (2001). Advances in Fusion Bonding Techniques for Joining Thermoplastic Matrix Composites: A Review. Compos. Part A Appl. Sci. Manuf..

[12] Van Ingen J.W., Buitenhuis A., Van Wijngaarden M., Simmons F. Development of the Gulfstream G650 Induction Welded Thermoplastic Elevators and Rudder. Proceedings of the International SAMPE Symposium and Exhibition (Proceedings).

[13] Villegas I.F., van Moorleghem R. (2018). Ultrasonic Welding of Carbon/Epoxy and Carbon/PEEK Composites through a PEI Thermoplastic Coupling Layer. Compos. Part A Appl. Sci. Manuf..

[14] Palardy G., Villegas I.F. Smart Ultrasonic Welding of Thermoplastic Composites. Proceedings of the the American Society for Composites-31st Technical Conference, ASC 2016.

[15] Jongbloed B., Teuwen J., Palardy G., Fernandez Villegas I., Benedictus R. (2020). Continuous Ultrasonic Welding of Thermoplastic Composites: Enhancing the Weld Uniformity by Changing the Energy Director. J. Compos. Mater..

[16] Troughton M.J. (2008). Handbook of Plastics Joining: A Practical Guide.

[17] Offringa A., Myers D., Buitenhuis A. Redesigned A340–500/600 Fixed Wing Leading Edge (J-Nose) in Thermoplastics. Proceedings of the 22nd International SAMPE Europe Conference.

[18] Gardinger G. Thermoplastic Composites Gain Leading Edge on the A380. https://www.compositesworld.com/articles/thermoplastic-composites-gain-leading-edge-on-the-a380.

[19] Thermoplastic Rudder and Elevator in G650 Empennage. https://www.gknaerospace.com/en/our-technology/2017/thermoplastic-rudder-and-elevator-in-g650-empennage/.

[20] Veldman S.L., Kortbeek P., Wölcken P.C., Kos H., Villegas J. Development of a Multifunctional Fuselage Demonstrator. Proceedings of the Aerospace Europe Conference.

[21] Omairey S.L., Sampethai S., Hans L., Worrall C., Lewis S., Negro D., Sattar T., Ferrera E., Blanco E., Wighton J. (2021). Development of innovative automated solutions for the assembly of multifunctional thermoplastic composite fuselage. Int. J. Adv. Manuf. Technol..

[22] Zweifel L., Zhilyaev I., Brauner C., Rheme M., Eckhard G., Bersier V., Glavaški S., Pfeiffer R. (2021). Experimental and Numerical Development on Multi-Material Joining Technology for Sandwich-Structured Composite Materials. Materials.

[23] MM-Welding®–Smart Joining of Lightweight Materials. https://www.bossard.com/ch-en/product-solutions/brands/welding-technology/mm-welding/.

[24] Engelschall M., Larsen L., Fischer F., Kupke M. Robot-Based Continuous Ultrasonic Welding for Automated Production of Aerospace Structures. Proceedings of the SAMPE Europe Conference.

[25] Don R.C., Gillespie J.W., McKnight S.H. (1997). Bonding Techniques for High Performance Thermoplastic Compositions. U.S. Patent.

[26] Brauner C., Nakouzi S., Zweifel L., Tresch J. (2020). Co-Curing Behaviour of Thermoset Composites with a Thermoplastic Boundary Layer for Welding Purposes. Compos. Adv. Mater..

[27] Zweifel L., Brauner C. (2020). Investigation of the Interphase Mechanisms and Welding Behaviour of Fast-Curing Epoxy Based Composites with Co-Cured Thermoplastic Boundary Layers. Compos. Part A Appl. Sci. Manuf..

[28] Lestriez B., Chapel J.P., Gérard J.F. (2001). Gradient Interphase between Reactive Epoxy and Glassy Thermoplastic from Dissolution Process, Reaction Kinetics, and Phase Separation Thermodynamics. Macromolecules.

[29] Lipatov Y.S., Alekseeva T.T. (2007). Phase-Separated Interpenetrating Polymer Networks. Phase-Separated Interpenetrating Polymer Networks.

[30] Teuwen J.J.E., Asquier J., Inderkum P., Masania K., Brauner C., Villegas I.F., Dransfeld C. Gradient Interphases between High Tg Epoxy and Polyetherimide for Advanced Joining Processes. Proceedings of the ECCM-18 Conference.

[31] Farooq U., Heuer S., Teuwen J., Dransfeld C. (2021). Effect of a Dwell Stage in the Cure Cycle on the Interphase Formation in a Poly(ether imide)/High Tg Epoxy System. ACS Appl. Polym. Mater..

[32] Van Krevelen D.W.W. (2009). Properties of Polymers.

[33] Tsiangou E., Teixeira de Freitas S., Fernandez Villegas I., Benedictus R. (2019). Investigation on Energy Director-Less Ultrasonic Welding of Polyetherimide (PEI)-to Epoxy-Based Composites. Compos. Part B Eng..

[34] Bruckbauer P. (2018). Struktur-Eigenschafts-Beziehungen von Interphasen Zwischen Epoxidharz und Thermoplastischen Funktionsschichten für Faserverbundwerkstoffe.

[35] Zweifel L., Brauner C., Teuwen J., Dransfeld C. (2022). In Situ Characterization of the Reaction-Diffusion Behavior during the Gradient Interphase Formation of Polyetherimide with a High-Temperature Epoxy System. Polymers.

[36] SikaPower^®^-1200. https://industry.sika.com/en/home/renewable-energies/wind-energy/blade-manufacturing/surface-finishingandrepair/sikapower-1200.html.

[37] 3M^TM^ Structural Adhesive SA9820. https://www.3m.com/3M/en_US/p/d/b40066524/.

[38] ARALDITE^®^ 2012. https://www.huntsman.com/products/araldite2000/araldite-2012.

[39] SikaPower^®^-1277. https://industry.sika.com/en/home/transportation/structural-adhesives/metal-adhesives/sikapower-1277.html.

[40] Phenoxy PKHH PELLETS. https://www.gabrielchem.com/product/pkhh-pellets/.

[41] Dynamic Fluid Compression Molding. https://www.compositesworld.com/cdn/cms/DFCMOverview.pdf.

[42] Huntsman Advanced Materials Develops a New Compression Molding Process. https://www.huntsman.com/about/advanced-materials/news/detail/12600/huntsman-advanced-materials-develops-a-new-compression.

[43] Alif N., Carlsson L.A., Boogh L. (1998). The Effect of Weave Pattern and Crack Propagation Direction on Mode I Delamination Resistance of Woven Glass and Carbon Composites. Compos. Part B Eng..

[44] Villegas I.F. (2019). Ultrasonic Welding of Thermoplastic Composites. Front. Mater..

[45] Palardy G., Villegas I.F. (2017). On the Effect of Flat Energy Directors Thickness on Heat Generation during Ultrasonic Welding of Thermoplastic Composites. Compos. Interfaces.

[46] Villegas I.F., Palardy G. (2017). Ultrasonic Welding of CF/PPS Composites with Integrated Triangular Energy Directors: Melting, Flow and Weld Strength Development. Compos. Interfaces.

[47] Fernandez Villegas I., Valle Grande B., Bersee H.E.N., Benedictus R. (2015). A Comparative Evaluation between Flat and Traditional Energy Directors for Ultrasonic Welding of CF/PPS Thermoplastic Composites. Compos. Interfaces.

[48] Da Silva L.F.M., Öchsner A., Adams R.D. (2018). Handbook of Adhesion Technology.

[49] Kupski J. (2020). Novel Adherend Laminate Designs for Composite Bonded Joints.

[50] Rajagopalan G., Gillespie J.W., McKnight S.H. (2000). Diffusion of Reacting Epoxy and Amine Monomers in Polysulfone: A Diffusivity Model. Polymer.

[51] Voleppe Q., Pardoen T., Bailly C. (2016). Interdiffusion and Phase Separation upon Curing in Thermoset-Thermoplastic Interphases Unravelled by the Characterization of Partially Cured Systems. Polymer.

[52] Villegas I.F. (2014). Strength Development Versus Process Data in Ultrasonic Welding of Thermoplastic Composites with Flat Energy Directors and its Application to the Definition of Optimum Processing Parameters. Compos. Part A Appl. Sci. Manuf..

[53] Schollerer M.J., Kosmann J., Völkerink O., Holzhüter D., Hühne C. (2019). Surface Toughening–A Concept to Decrease Stress Peaks in Bonded Joints. J. Adhes..

[54] Brauner C., Küng M., Arslan D., Maurer C. (2021). Fused Filament Fabrication Based on Polyhydroxy Ether (Phenoxy) Polymers and Related Properties. Polymers.

